# Validation of a Dumbbell Body Sway Test in Olympic Air Pistol Shooting

**DOI:** 10.1371/journal.pone.0096106

**Published:** 2014-04-22

**Authors:** Daniel Mon, Maria S. Zakynthinaki, Carlos A. Cordente, Antonio Monroy Antón, David López Jiménez

**Affiliations:** 1 Universidad Politécnica de Madrid. Sports Department, Madrid, Spain; 2 Applied Mathematics and Computers Laboratory, Technical University of Crete, Greece; 3 Universidad Francisco de Vitoria. Sports Department. Madrid, Spain; 4 Universidad Autónoma de Chile. Profesor investigador. Santiago de Chile, Chile; University of Rome, Italy

## Abstract

We present and validate a test able to provide reliable body sway measurements in air pistol shooting, without the use of a gun. 46 senior male pistol shooters who participated in Spanish air pistol championships participated in the study. Body sway data of two static bipodal balance tests have been compared: during the first test, shooting was simulated by use of a dumbbell, while during the second test the shooters own pistol was used. Both tests were performed the day previous to the competition, during the official training time and at the training stands to simulate competition conditions. The participantś performance was determined as the total score of 60 shots at competition. Apart from the commonly used variables that refer to movements of the shooters centre of pressure (COP), such as COP displacements on the X and Y axes, maximum and average COP velocities and total COP area, the present analysis also included variables that provide information regarding the axes of the COP ellipse (length and angle in respect to X). A strong statistically significant correlation between the two tests was found (with an interclass correlation varying between 0.59 and 0.92). A statistically significant inverse linear correlation was also found between performance and COP movements. The study concludes that dumbbell tests are perfectly valid for measuring body sway by simulating pistol shooting.

## Introduction

Amongst all Olympic shooting modalities, air pistol is one where the static component plays a crucial role. Air pistol shooting is a “precision and accuracy” modality. It demands exceptional concentration and a strong mental approach, as the slightest uncontrolled movement can lead to failure. The target dimension is very small (the area of maximum score of 10 points has a diameter of 11.5 mm±0.1 mm) in comparison to the shooting distance of 10 m [1]. Angular deviations as small as 0,066° can, therefore, lead to important loss of points in high level pistol shooting.

Although many are the factors that influence performance in Olympic air pistol shooting, the ability to stabilize the gun seems to play the most important role [2], [3], [4]. A shooter’s skill in minimizing the movements of the gun is determined by static balance [5], muscular tremor [6], coordination between shooting and trigger pressing time [7], [8], experience and training [9] as well as physical condition [10]. Even though no specific body morphology seems to play role in Olympic shooting, the Olympic shooters are in general shorter and heavier than athletes of different disciplines [11].

There seems to be a consensus that the ability to stabilize the gun and consequently performance, is controlled by the movements of the body’s center of pressure (COP) [12], [13], [14]. It has been shown [3] that the body’s movements along the Y axis are related to lateral movements of the gun along the X axis, and that the gun movements along the Y axis are related to arm movements caused by variations in the position of the shoulder. Significant correlations have been reported between performance and COP movements in novice shooters [2], [15]. The degree of influence of this variable varies, however, amongst the different studies.

For maximum stabilization of the gun, maximum body movement control is therefore required by the shooter. A consensus exists in the scientific literature as well as amongst the specialized coaches that better static balance leads to higher levels in performance [4], [16], [17], [18], [19].

The gun movements are currently measured by use of optoelectronic system, like NOPTEL [20] or SCATT [12]. However, the validity of such systems has been questioned [21]. The COP movements are measured by use of widely accepted force platforms. Various variables are used for the measurement of the COP movements, such as the total area of COP displacement [18] or the maximum distances run by the COP on the X and Y axes [15] or the average or maximum velocities on the X and Y axes [22], [23], with the later being the most used variables according to [20].

All existing studies related to measurements of gun or COP movements are based on experimental protocols that use an actual gun and are performed under laboratory or training conditions. No study can be found that is based on data recorded under actual competition conditions, although this is the only condition under which performance can be accurately measured. Furthermore, a valid and reliable test for measurements of the COP movements where the gun is substituted by a dumbbell of similar weight would allow measurements to be carried out anywhere (such as in schools or sport centers for example, where the use of a gun is prohibited) facilitating and augmenting the possibility for young talent detection.

The objective of the present study was to design and validate a test where measurements of the COP movements in Olympic air pistol shooting can be carried out without the use of an actual air pistol.

## Materials and Methods

### Ethics Statement

The Ethical Board of the Spanish Team Sports Association approved the experimental design of the study. The informed consent document that all the participants signed before data collection was also approved by the Ethical Board of the Spanish Team Sports Association. We confirm that our research meets the highest ethical standards for authors and co-authors. The study was performed following the guidelines of the Declaration of Helsinki, last modified in 2008.

The authors certify that the present research was carried out in the absence of any financial, personal or other relationships with other people or organizations that could inappropriately influence, or be perceived to influence, the presented work and lead to a potential conflict of interest.

### Participants

The study is based on COP movement data of 46 senior male pistol shooters who participated in Spanish air pistol championships (the senior category is referred to ages between 21 and 54 years old [1]. According to the regulations of the Spanish Federation of Olympic Shooting (2012) eligibility to compete required a previously obtained minimum score of 510 points in air pistol in other national competitions [1]. It should be mentioned here that current world record is 594 points (with the maximum being 600 points–60 shots).

The participants profile is shown in table 1.

**Table 1:** Participants profile, mean values ± standard deviation.

Age (years)	42,7	±	10,67
Height (m)	1,75	±	0,07
Weight (Kg)	87,38	±	13,3
BMI (kg/m^2^)	28,6	±	4,6
Experience (years)	11,92	±	8,9
Training (hours/week)	5,7	±	5,64
Performance	548,2	±	13,7

### Experimental Protocol

The protocol consisted of two static bipodal balance tests.

During the first test, shooting was simulated by use of a 1.5 kg dumbbell. This weight corresponds to the maximum official gun weight, as established by the Real Federación Española de Tiro Olímpico [1]. The test was performed following the criteria of the study of Gulbinskiene. and Skarbalius [5] ensuring the similarity of the technique with the actual shooting gesture.

For the second test the participants used their air pistol to shoot. The maximum dimensions of all pistols used were 0.42×0.2×0.05 m with a minimum trigger pressing weight of 0.5 kg, as stated by article 8.16.0 of the pistol regalement [24]. The use of the shooters’ own pistol was preferred in order to guarantee specificity and to allow adaptability to the shooters’ individual characteristics and the subsequent comparison with their performance.

Both tests were performed the day previous to the competition, during the official training time and at the training stands to simulate competition conditions.

The participants initial position was their natural shooting position. In order to standardize the tests, the guidelines of Hawkins & Sefton [23] were followed, in relation to the feet distance during the test. The maximum feet distance was therefore kept between 0.3 and 0.6 m, as for such distance no differences in the COP movements have been reported.

Both tests were repeated three times, as suggested by Pinsault & Vuillerme [25], in order to guarantee accurate measurements of the COP parameters as well as reproducibility of the test, for each participant. The protocol also took into account the limited available time (8 hours) of the official training previous to competition, trying at the same time to simulate the actual pace and rhythms during competition (60 shots in 90 minutes, 90 seconds between shots).

The shooting simulations took place under luminosity of 1240 luxon. The distance to the target was 10 m and the height of the target’s centre was 1.4 m (measured from the level of the shooting stand). To visually complete the simulation of a shot, the targets used were official paper targets.

Reinkemeier et al [4] report that the shooting time oscillates between 6 and 10 seconds, depending on the shooter, with 8 seconds being the optimal time. Some shooters, however, exceed this time. For this reason and in order to respect the specific time of each shooter and optimize the data recording of such a specific test, the duration of each test was decided to be 15 seconds.

Each recording started the moment the shooter was ready and holding the gun/dumbbell ready to shoot/simulate shooting. A resting period of 1.5 minutes was allowed between test repetitions. The shooters performance was calculated as the total score of 60 shots at competition.

### Apparatus-equipment

A portable force platform (Kistler 9286AA) was used to record b the COP movements on the X (anterior-posterior) and Y (medium-lateral) axes at a frequency of 100 Hz. The shooters performance was measured by use of official paper targets, according to the International Shooting Sport Federation (ISSF) Rules and Regulations (Edition 2009) and as provided by the referees of the Spanish Olympic shooting federation after the competition. The luminosity was measured with a HT307 luxmeter.

### Statistical Analysis

The Kolmogorov-Smirnov test was used to determine the goodness of fit to the normal distribution of the variables. As all calculated p-values were greater than the level of significance which was set equal to p = 0.05, the test failed to reject the null hypothesis that the variables were normally distributed. The only exception was found for the variable of training hours, where a value of p<0.01 was calculated, revealing that the number of training hours did not follow a normal distribution. This result was, however, expected as the group of participants included elite shooters whose training involves many more hours per week. Linear correlations were calculated between performance and COP movements or morphology of the participants. In order to analyze the concurrent validity of the variables for both tests, Pearson interclass correlations were calculated. The level of significance was set at p = 0.001.

The statistical analysis of the variables was performed using SPSS PASW Statistics 17. The calculation of the displacements, velocities, areas and angles was carried out by use of the mathematical package Matlab R2009a.

### Variables

For the purposes of the present study the following variables were analysed regarding the participants profile: Weight (kg), height (m), experience (years), training (hours per week) and performance over 60 shots.

Referring to COP movements, the following variables were analysed (table 2): Maximum COP displacements on the X and Y axes, total area of COP displacement, average and maximum COP velocities on the force platform plane, average and maximum COP velocities on the X and Y axes.

**Table 2:** Mean ± standard deviations of the variables referring to the participants COP movement.

	Pistol	Dumbbell
**Max. displ. X**	14.96±4.15	15.15±4.03
**Max. displ. Y**	7.42±1.75	7.94±2.6
**Principal axis**	14.85±3.88	15.33±3.41
**Secondary axis**	10.52±3.44	10.34±3.19
**Angle**	3,39±10,39	4,45±9,75
**Total area**	121.95±80.06	121.21±66.64
**Aver. velocity X**	16.15±2.27	16.52±2.42
**Max. velocity X**	75.01±11.24	75.98±11.95
**Aver. velocity Y**	24.07±3.67	24.11±3.54
**Max. velocity Y**	112.10±17.72	109.91±16.82
**Aver. COP velocity**	31.87±4.50	32.16±4.46
**Max. COP Velocity**	115.91±18.30	113.88±17.38

The units are: COP displacements, m*10^−3^; angle, degrees; area, m*10^−6^; COP velocities, m/sec*10^−3^.

**Figure 1:**
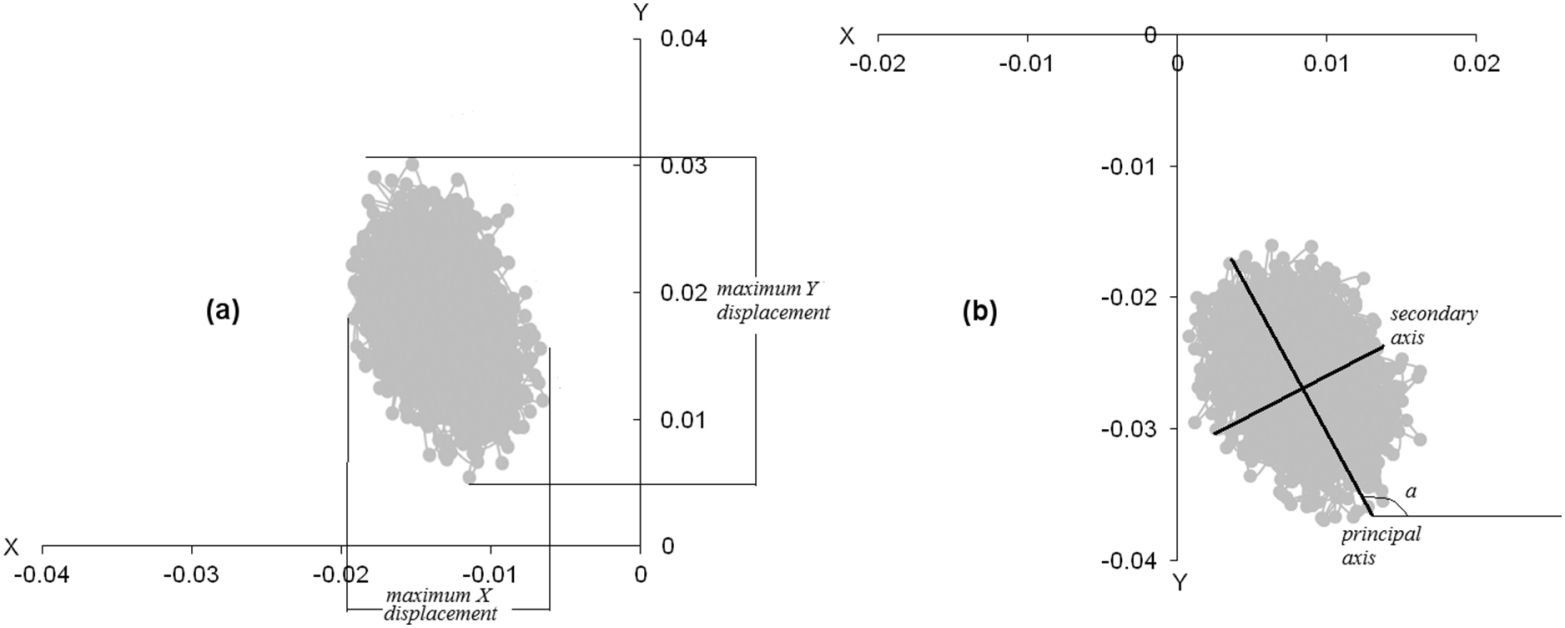
Examples of body sway graph showing the COP movements of the same participant, as recorded on the force platform plane. (a): By use of a pistol, (b) by use of a dumbbell simulating a pistol. The total COP movement can be assumed to be included within an ellipse, the principal axis of which is rotated in respect to X axis.

We also analyzed the length of the principal and the secondary axis of the ellipse that best fitted each participant’s COP data, as well as the angle between the principal axis of the ellipse and the X axis (figure 1).

## Results

Significant inverse correlations (shown in table 3) were found between performance and maximum COP displacement on the X axis in pistol shooting (*F_1,43_ = 4,92; P<0.05*), and also between performance and length of the COP ellipse principal axis in dumbbell shooting (*F_1,43_ = 4,11; P<0.05*). Performance was also found to be significantly correlated with experience (*F_1,43_ = 7.18; P<0.05*) and training (*F_1,43_ = 11.51; P<0.01*). For the rest of the variables no significant correlations were found.

**Table 3:** Linear correlations between performance and a) length of the COP ellipse principal axis in dumbbell shooting, b) maximum COP displacement on the X axis in pistol shooting, c) experience and d) training.

	r	r^2^ corrected	CV% = SD/M×100	significance
COP ellipse principal axis, dumbbell	0,32	0,1	2,39	0,03*
Max. COP X displacement, pistol	0,3	0,09	2,16	0,04*
Experience	0,38	0,12	2,34	0,01*
Training	0,46	0,19	2,24	0,001**

Coefficient of variation = SD/M×100.

*Level of significance p<0.05,

**Level of significance p<0.01.

The analysis of Pearson interclass correlations for the variables that refer to the movement of the COP for both tests, revealed statistically significant results for all variables (table 4).

**Table 4:** Pearson interclass correlations for the variables that refer to the movement of the COP, for the data of both tests.

Variable	Pearson correlation coefficient
**Max. Displ. X**	0,60
**Max. Displ. Y**	0,64
**Principal axis**	0,59
**Secondary axis**	0,59
**Angle**	0,68
**Total area**	0,73
**Aver. velocity X**	0,87
**Max. Velocity X**	0,75
**Aver. velocity Y**	0,92
**Max. Velocity Y**	0,81
**Aver. COP Velocity**	0,91
**Max. COP Velocity**	0,85

Level of significance p<0.001.

## Discussion and Conclusion

Static balance seems to play a very important role in stabilizing the gun in pistol shooting [4]. As the use of a gun is essential in Olympic shooting, the majority of the studies use a gun to evaluate the shooters static balance by measuring the COP movements of the shooter’s body [3]. The need of a pistol can, however, be a drawback, especially in places like schools or sport centres where the use of a gun is prohibited. There is, therefore, the need of the development (and validation) of a test that will provide reliable body sway measurements without the use of a gun.

In addition, the validation and use of a standard test that does not require the use of a pistol would provide an easy and fast way for the evaluation of a subject’s COP movements by use of inexpensive materials, such as dumbbells. Expensive force platforms could be also replaced by other validated and widely distributed devices, like for example Nintendo wii balance boards [26].

The present study compared body sway data recorded during shooting with a pistol and shooting with a dumbbell that simulated a pistol. Regarding the variables that were selected in order to measure and evaluate the body sway of the shooters, the present study analyzed all variables that are used in the literature (such as COP displacements on the X and Y axes, maximum and average COP velocities and total COP areas). With the aim to present a complete analysis, the present study extended the variables including information regarding the axes of the COP ellipse (lengths and angle in respect to X).

The analysis of the recorded data showed a strong correlation between the two tests regarding all variables, with Pearson interclass correlations varying between 0.59 and 0.92. More specifically, the correlations referring to the movements of the COP were moderate to strong, varying from 0.59 to 0.73, while the correlations referring to the velocities of the COP movements were very strong, varying from 0.75 to 0.92. This justifies the validity of the dumbbell test [27].

In addition and in accordance with existing studies [2], [13], [14], the analysis presented here showed statistically significant correlations between COP movements and performance. Even though not all COP variables were found to be significantly correlated with performance, a tendency of an inverse linear corelation was found regarding the length variables used to characterize the movements of the COP. This result justifies specific shooting books that report that shooters with less body sway tend to perform better [4], [16]. The importance of this result becomes clear when one takes into account that the coefficient of variation found (ranging from 2.13 to 2.39, otherwise not so significant) is equivalent to 13 or 14 points of the final competition score (600 points being the maximum score). This difference in points corresponds to an average of 52–57 positions in the final ranking, according to an analysis of the Olympic shooting world championships from 1998 to date [28].

We conclude that specific body sway tests like the one described in the present study can be used to evaluate a subject’s static balance, by using a dumbbell to simulate air pistol shooting. Future studies are recommended in order to confirm the validity and reliability of the test with the dumbbell in other shooting categories and/or female categories.

## References

[1] RFEDETO (2012b) Reglamento Técnico General para todas las Modalidades de Tiro (2009 ed.). Madrid: Real Federación Española de Tiro Olímpico.

[2] Mononen K, Konttinen N, Viitasalo J, Era P (2007) Relationships between postural balance, rifle stability and shooting accuracy among novice rifle shooters. Scand J Med Sci Sports 17(2): 180–185. 17394480 10.1111/j.1600-0838.2006.00549.x

[3] Pellegrini B, Schena F (2005) Characterization of arm-gun movement during air pistol aiming phase. J Sports Med Phys Fitness 45(4): 467–475. 16446677

[4] Reinkemeier H, Bühlmann G, Konietzny A (2006) Olympisches Pistolen-Schieβen: Technik, Training, Taktik, Psyche, Waffen; ein Lehr- und Übungsbuch zum sportlichen Schieβen mit der Luftpistole, der Sportpistole und der freien Pistole. MEC High Tech Shooting Equipment.

[5] Gulbinskiene. V, Skarbalius A (2009) Peculiarities of investigated characteristics of lithuanian pistol and rifle shooterś training and sport performance. Ugdymas Kuno Kultura 73: 21–27.

[6] Lakie M (2010) The influence of muscle tremor on shooting performance. Experimental Physiology 95(3): 441–450. 19923157 10.1113/expphysiol.2009.047555

[7] Viitasalo J, Era P, Konttinen N, Mononen K, Mononen H, et al (1999) The posture steadiness of running target shooters of different skill levels. Kinesiology 31: 11.

[8] Zatsiorsky V, Aktov A (1990) Biomechanics of highly precise movements: the aiming process in air rifle shooting. J Biomech 23: 35–41. 10.1016/0021-9290(90)90039-6 2081743

[9] Goonetilleke RS, Hoffmann ER, Lau WC (2009) Pistol shooting accuracy as dependent on experience, eyes being opened and available viewing time. Applied Ergonomics 40(3): 500–508. 18992872 10.1016/j.apergo.2008.09.005

[10] Krasilshchikov O, Zuraidee E, Singh R (2007) Effect of general and auxiliary conditioning on specific fitness of young pistol and rifle shooters. Asian J. Exerc. Sport Sci 4: 01–06.

[11] Belinchon F (2010) Estudio médico deportivo de las modalidades de tiro olímpico. Universidad Complutense de Madrid: Madrid.

[12] Ball KA, Best RJ, Wrigley TV (2003) Inter- and intra-individual analysis in elite sport: Pistol shooting. J Appl Biomech 19(1): 28–38.

[13] Mason B, Cowan L, Gonczol T (1990) Factors affecting accuracy in pistol shooting. Excel 6: 2–6.

[14] Viitasalo J, Era P, Mononen H, Norvapalo K, Rintakoski E (1998) Effects of footwear on posture control of running target shooters. Int J Sports Sci Coach 3(2): 3–6.

[15] Era P, Konttinen N, Mehto P, Saarela P, Lyytinen H (1996) Postural stability and skilled performance–a study on top-level and naive rifle shooters. J Biomech 29(3): 301–306. 8850636 10.1016/0021-9290(95)00066-6

[16] Mon D (2006) Objetivos y ventajas de la preparación física en el tiro olímpico: una primera aproximación. Tiro Olímpico 60: 18–21.

[17] Aalto H, Pyykko I, Ilmarinen R, Kahkonen E, Starck J (1990) Postural stability in shooters. ORL J. Otorhinolaryngol. Relat. Spec 52(4): 232–238. 10.1159/000276141 2392286

[18] Herpin G, Gauchard GC, Lion A, Collet P, Keller D, et al (2010) Sensorimotor specificities in balance control of expert fencers and pistol shooters. J Electromyogr Kines 20(1): 162–169. 10.1016/j.jelekin.2009.01.003 19217310

[19] Konttinen N, Lyytinen H, Viitasalo J (1998) Rifle balancing in precision shooting: behavioral aspects and psychophysiological implication. Scandinavian Journal of Medicine & Science in Sports 8(2): 78–83. 9564711 10.1111/j.1600-0838.1998.tb00172.x

[20] Hawkins R (2011) Identifying mechanic measures that best predict air-pistol shooting performance. Int J Perform Anal Sport 11(3): 499–509.

[21] Zanevskyy I, Korostylova Y, Mykhaylov V (2010) Shot Moment in Optoelectronic Training in the Air-Pistol Shooting. Int J Sport Sci and Engin 4: 67–78.

[22] Su FC, Wu WL, Lee WD (2000) Stance Stability in Shooters. J Med Biol Eng 20(4): 187–192.

[23] Hawkins RN, Sefton JM (2011) Effects of stance width on performance and postural stability in national-standard pistol shooters. J Sports Sci 29(13): 1381–1387. 21916795 10.1080/02640414.2011.593039

[24] RFEDETO (2012a) Reglamento Técnico Especial para Pistola. Madrid: Real Federación Española de Tiro Olímpico.

[25] Pinsault N, Vuillerme N (2009) Test-retest reliability of centre of foot pressure measures to assess postural control during unperturbed stance. Med Eng Phys 31: 276–286. 18835738 10.1016/j.medengphy.2008.08.003

[26] Clark RA, Bryant AL, Pua Y, McCrory P, Bennell K, et al (2010) Validity and reliability of the Nintendo Wii Balance Board for assessment of standing balance. Gait & Posture 31(3): 307–310. 20005112 10.1016/j.gaitpost.2009.11.012

[27] Fleiss JL (1986) The design and analysis of clinical experiments: Wiley Online Library.

[28] ISSF (2013) issf-sports.org. Retrieved September 27, 2013 from http://www.issf-sports.org/results.ashx.

